# Criminal law-based copyright protection with entrepreneurial spirit

**DOI:** 10.3389/fpsyg.2022.944122

**Published:** 2022-09-08

**Authors:** Wenjing Wang

**Affiliations:** School of Law, Tsinghua University, Beijing, China

**Keywords:** entrepreneurial spirit, enterprise copyright, criminal law protection, economic growth, enterprise innovation

## Abstract

This study aims to optimize the enterprise criminal law-based copyright protection. This exploration discusses the role of the entrepreneurial spirit (ES) in criminal law-based copyright protection. To study the relationship between ES and criminal law-based copyright protection, the concepts of ES, criminal law-based copyright protection, and enterprise innovation are given. Next, by collecting literature, hypotheses are put forward. They include the relationship between ES and enterprise innovation, ES and the criminal law-based copyright protection, and the intermediary role of ES in the criminal law-based copyright protection and economic growth. Then, relevant models are established. Finally, the hypotheses are tested through experiments and empirical analysis, and the model is regressed to test the experimental data’s robustness and the scale’s reliability and validity. The empirical analysis shows that: (1) the significance of ES under the 1% index is greater than 0. It indicates that the higher the managers’ ES is, the greater the enterprise innovation is. (2) The significance of criminal law-based copyright protection on ES under the 1% index is greater than 0 and the regression coefficient is 0.59. This shows that criminal law-based copyright protection has a significant positive impact on ES. (3) Under the l% index, the significance of ES on economic growth is greater than 0 and the regression coefficient is 0.63. It shows that ES mediates the relationship between criminal law-based copyright protection and economic growth. Therefore, strengthening criminal law-based copyright protection improves the ES and leads to faster enterprise and regional economic development. Therefore, the state should pay attention to criminal law-based copyright protection to encourage innovation to promote enterprise development. This exploration studies the relationship among ES, economic growth, enterprise innovation, and criminal law-based copyright protection. The finding provides a theoretical reference for criminal law-based copyright protection.

## Introduction

At present, China’s economy is in a new stage. The traditional means of economic growth need to be further optimized, and economic transformation must be strengthened. Independent innovation is key to enterprise work efficiency (Badii and Sharif, 2020). Relevant research shows that the strength of enterprise innovation will be restricted by various factors, including external and internal factors. External factors include the strength of criminal law-based copyright protection, the complexity of the bank loan process, and the government’s macroeconomic regulation and control ability (Li et al., 2021). Internal factors include enterprise management, employee innovation, and enterprise sustainability (Zhang et al., 2022).

The exogenous (represented by Solow) and endogenous (represented by Lucas and Romer) economic growth theories both believe that technological innovation and diffusion are the main driving forces of economic growth. Copyright protection highlights technological innovation and diffusion (Liu, 2020). The comprehensive opening-up policy and globalization have introduced fierce international competition represented by high-tech giants into the Chinese market. The impact of copyright protection on economic growth has gradually expanded to the global perspective (Megías et al., 2020). In terms of international trade, since the World Trade Organization (WTO) reached the agreement on Trade-related Copyright Protection, the relevance between copyright policy and international trade policy has intensified and has become a key institutional factor affecting a nation’s international trade (Cui and Xu, 2020). However, the initial resource consumption of innovation activities is large, and the final innovation results are uncertain. Therefore, society should have better expectations to make the scientific and technological innovation environment have a high degree of vitality release. The emergence of reasonable social expectations is inseparable from copyright’s criminal law protection system (Idewele et al., 2021). In recent years, because of its great significance and the increasing attention of the state, innovation has successfully turned ES into the focus of discussion (Rustiana et al., 2021).

Thus, the existing research on enterprise innovation mainly analyzes the impact of the legal system (external factor) and enterprise management (internal factor). However, whether ES positively affects enterprise innovation is not investigated. Moreover, most scholars only study the interaction between ES and economic growth without further discussing strengthening the ES. In the research and application of Linear Regression Model (LRM), non-normal distribution assumption, non-LRM, and fusion model are the research focus. First, in reality, the relationship between non-linear variables is absolute and universal, while the relationship between linear variables is relative and unique. Therefore, the non-LRM is irreplaceable. So far, there is no non-LRM modeling method. Usually, the non-LRM is transformed into an LRM with the idea of non-linear linearization. Based on this, this exploration first gives the concepts of ES, criminal law-based copyright protection, and enterprise innovation. Next, combined with the literature, the relationship among them is analyzed, and hypotheses and models are put forward. Finally, the hypothesis is verified by setting experiments. This exploration aims to study the relationship between criminal law-based copyright protection and ES to provide an important reference for optimizing the provisions of criminal copyright law.

## Literature review

### Entrepreneurial spirit

At first, scholars think entrepreneurs are only responsible for managing employees and capital, paying attention to the development of enterprises, and handling daily production tasks (Abdelkafi and Refas, 2021). Therefore, in neoclassical economics, scholars always take the enterprise as a unified whole to calculate its costs and benefits. They did not realize that entrepreneurs’ special ability or ES is an important production factor. Only recently, it was found that ES will significantly impact the enterprise’s cost and income function and further affect its various decisions (Yeganegi et al., 2021). With the gradual progress of economic theory, scholars gradually realize that ES is not only a critical intangible wealth of enterprises but also a key factor of production. The concentration of these production factors in each region will significantly affect the local economic development capacity (Mittal and Raghuvaran, 2021). Some scholars have proved that the higher the ES of a country or region is, the faster the region’s economic development is (Setiawan et al., 2021). Although many studies have repeatedly emphasized the importance of ES, there is still no comprehensive and clear definition. According to most scholars, the ES can be divided into three aspects: (1) the judgment of entrepreneurs: The ES is the ability of entrepreneurs to make judgmental decisions on allocating scarce resources under uncertain conditions; (2) keen vigilance: the ES is the alertness of entrepreneurs to find the unbalanced information and arbitrage opportunities of the market in the unbalanced state to make the market transition from the unbalanced state to the equilibrium state; and (3) ability to take risks and deal with uncertainty: some scholars hold that the difference between employees and entrepreneurs mainly lies in income uncertainty. The biggest difference between entrepreneurs and ordinary workers lies in their ability to take risks and deal with uncertainty (Derclaye, 2020).

**FIGURE 1:**
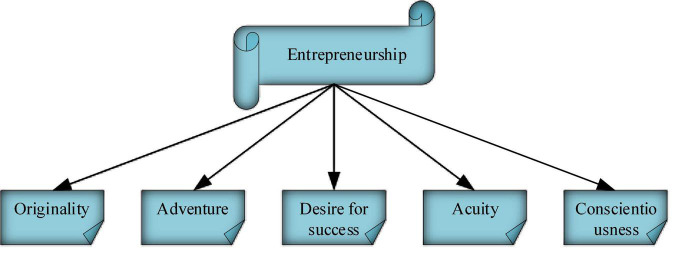
Elements of ES.

Figure 1 shows the specific elements of ES.

### Copyright protection

Copyright refers to the sum of the owners or some specific institutions’ personal rights and property rights for literary, artistic, architectural, photographic, cinematographic, painting, computer software, and works related to the law (Fomin, 2020). As for the definition of works, China’s *Copyright Law* stipulates that works are intellectual achievements with originality and innovation expressed in some physical form (Greene et al., 2020).

Copyright is a major measure of enterprise development (ED) and highlights enterprise competitiveness. It will also have a certain impact on the management system and innovative means of enterprises. Generally, copyright value is reflected in patent publication, namely, the strength of enterprise innovation. On one hand, patents protect enterprise innovation through criminal law. On the other hand, they will have a certain positive impact on enterprise innovation activities. Enterprise patent level can promote enterprise competition by collecting favorable resources according to the industrial situation. It is divided into patent publication, utilization, protection, and management level (Martin and Minnichelli, 2020).

### Enterprise innovation

**FIGURE 2:**
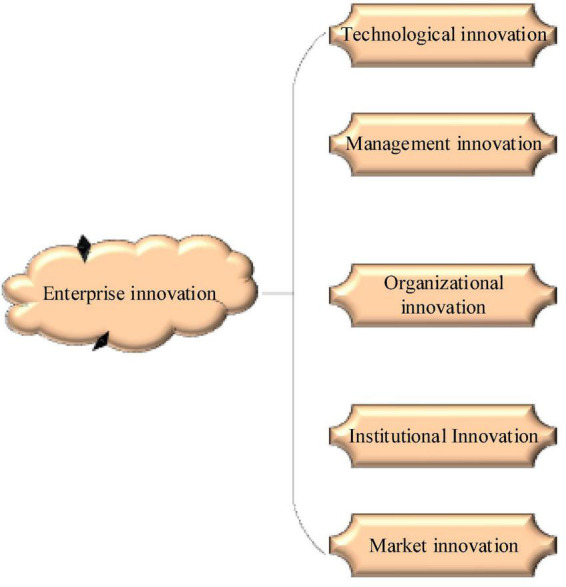
Specific contents of enterprise innovation.

The famous Austrian American economist Joseph Alois Schumpeter put the innovation concept in the early 20th century. Innovation is believed to be the combination of production factors and conditions introduced into the production system, leading to the change in production function (Kovalchuk et al., 2020). Innovation can be divided into five aspects: (1) creating new goods; (2) adopting new methods, that is, new means of production; (3) building new markets; (4) grasping the new source of raw material supply; and (5) completing the new organization of the enterprise (Fridhi, 2021). According to the popular views in modern management circles, Figure 2 shows the detailed division of enterprise innovation.

In particular, the patent publication is the fundamental link of the enterprise patent level. It is the static patent resources obtained by enterprises. Therefore, it is crucial to study enterprises’ patent publication ability to improve innovation and competition (Kacperska and Ukasiewicz, 2020). Building an innovation system and continuously adopting innovation strategies can provide strategic protection for efficient economic development and cooperation between the economy and society. The main body of innovation is people and the main body of enterprise innovation is entrepreneurs. Entrepreneurial spirit (ES) is key to promoting enterprise innovation activities and operation mode optimization (Absalyamova et al., 2020).

The relevant research on the relationship between ES and copyright protection is based on enterprise innovation and economic growth. It studies the relationship between ES and copyright protection by taking enterprise innovation and economic growth as intermediary factors. Hence, ES and copyright protection will not only affect each other but also can influence one another through intermediary factors that cannot be ignored and can become a powerful tool for studying their relationship. In addition, the improvement of copyright protection has a positive impact on ES, and the improvement of ES will also positively impact the innovation of enterprises and economic growth. Therefore, based on the initial results, this work will further study the relationship between ES and copyright protection.

### Analysis of the relationship between enterprise innovation, entrepreneurial spirit, and criminal law-based copyright protection

First, enterprise innovation requires substantial long-term investment. There is a great demand for funds. Investors are more confident about the enterprise innovation return in areas with criminal law-protected copyright. They are more willing to increase investment in enterprise Research and Development (R&D) projects (Vitiuk, 2020). This greatly eases the financing constraints and extends the enterprise R&D investments. Second, enterprise R&D achievements are less likely to be plagiarized under criminal law-protected copyright. Enterprises are more willing to disclose patent information, thus reducing the cost of information queries. It helps other enterprises complete follow-up R&D, forming a benign industry innovation cycle and increasing overall industrial R&D enthusiasm. Finally, the innovative achievements are highly exclusive in criminal law-based copyright-protected areas, thus reducing patent infringement or imitation. Thus, it ensures that the huge investment in R&D can be rewarded from the monopoly profits of the product. In case of an infringement, the injured party will be given huge compensation. These can further promote adventurous entrepreneurs to engage in high-risk and high-yield investment activities, such as enterprise innovation. In addition, entrepreneurs are the main leaders and guide the direction and strength of ED. The higher the ES, the larger the investment in enterprise innovation. Therefore, to some extent, ES and copyright protection can impact the investment in enterprise innovation.

## Research methodology

### Proposal of research hypothesis

#### Entrepreneurial spirit and enterprise innovation

The process of entrepreneurs’ research on new means of production is called innovation. This means of production will complete “creative destruction” from the inside and unprecedentedly combine production factors and production conditions into the new production system to complete “creative accumulation” (Bu and Cuervo-Cazurra, 2020). ES affects the innovation activities of enterprises in two ways. First, the risk characteristics of innovation correspond to the preferences of entrepreneurs willing to take risks. In this state, innovation has the characteristics of high risk and high return, which is in line with managers’ preference for the ES. This preference will encourage enterprises to increase R&D investment in high-risk and high-return projects (Rb et al., 2020). Next, entrepreneurs with keen insight can find innovation opportunities and organize innovative production. Only entrepreneurs sensitive to innovation opportunities and management can perceive the internal and external environment changes and constantly promote innovative production to strengthen enterprises’ innovation ability (Xin et al., 2020).

With the enrichment and expansion of its concept, researchers from finance, management, psychology, economics, and other disciplines believe that ES is not a general concept. They interpret ES from different levels, such as individual, team, and organization, and believe that different levels of ES will have distinct effects on enterprise innovation performance.

Entrepreneurs, a group of initiative talents, can thoroughly understand their work roles. They actively improve the enterprise innovation performance, thanks to their innovation spirit, adventurous spirit, and other ES. Shin and Park (2019) analyzed the relationship between entrepreneurs’ mixed value orientation, ES, and enterprise innovation performance. The results showed that entrepreneurs’ mixed value orientation positively affected enterprise innovation performance. ES promoted the integration of mixed value orientation and innovation performance (Shin and Park, 2019). Efimushkin and Efimushkina (2020) argued that ES included the ability to innovate, create natural technology, and integrate technological innovation (Efimushkin and Efimushkina, 2020). Gurau and Dana (2020) contended that ES included the ability to obtain resources, adapt to adjustment, and entrepreneurial tendency, which would play an important role in creating enterprises (Gurau and Dana, 2020). Bojica and Fuentes (2012) found that ES and knowledge acquisition positively impacted technological innovation performance. The amount of knowledge acquisition was determined by the knowledge resources of enterprise teams (Bojica and Fuentes, 2012). Ndemezo and Kayitana (2018) claimed that the background (education and experience) of entrepreneurial team members and the motivation of senior managers significantly contribute to the innovation performance of manufacturing companies (Ndemezo and Kayitana, 2018).

Based on this, the following hypothesis is put forward:

**H1:** The innovation strength of enterprises increases with the continuous improvement of entrepreneurs’ ES.

#### Criminal law-based copyright protection and entrepreneurial spirit

Entrepreneurs are the top managers of enterprises. Therefore, for the future planning and operation of enterprises, entrepreneurs should play their guiding role, put forward relevant development strategies, and point out the direction for the company. However, to complete the above work, entrepreneurs must learn to tap the unbalanced data in the industry environment and formulate relevant plans to grasp the opportunities. Moreover, they should also learn to take diagnostic means to schedule rare resources reasonably. Besides, entrepreneurs must obtain more external environmental information to realize the ED strategy. Notably, the entrepreneurs’ abilities are determined by themselves and restricted by the institutional environment (Bakhramovna, 2021). Criminal law-based copyright protection impacts the product R&D. The importance, thoroughness, and patent scope of copyright are significant for commercializing inventions. The new information creation through the discovery of new knowledge and new technology and the information asymmetry caused by the market’s development provide entrepreneurs opportunities to improve their sensitivity and initiative and release their ES (Makarenko, 2020). The risk characteristics of innovation are consistent with the innovation preference of entrepreneurs. However, the uncertainty of innovation results also makes entrepreneurs face the possibility of failure. Whether an individual can agree to take risks is inseparable from the balance between the cost of failure and the income of success. The high-intensity criminal law-based copyright protection reduces the possibility of misappropriating innovative technological products and temporarily protects the legal monopoly compensation. Even if the copyright is infringed, it can generate higher compensation, which ensures the status of innovative products from the institutional level. Meanwhile, it also strengthens entrepreneurs’ adventurous spirit and desire for success to improve their ES. In China, criminal law-based copyright protection focuses on protecting the legitimate rights and interests of knowledge creators. It requires creators to disclose the content of achievements to society and give the public the thinking of re-innovation and optimization. The copyright owner ensures the correct use of copyright and enhances labor production efficiency by transforming copyright into goods. Thereby, it protects and stimulates entrepreneurs’ responsibility to serve society and cultivates entrepreneurs’ ES.

Based on this, the following hypothesis is put forward:

**H2:** The strength of criminal law-based copyright protection has a significant positive impact on ES.

#### The intermediary role of entrepreneurial spirit

As one of the important resources under the background of theoretical economy, criminal law-based copyright protection has gradually developed into the production basis of new competition. Strengthening the criminal law-based copyright protection can protect copyright owners’ legitimate rights and interests from infringement. When copyright owners put innovative technologies into the market, they can obtain high monopoly benefits. Doing so promotes all industries’ development capacity and gives other peer enterprises greater competitive pressure (Khurana and Dutta, 2021). The pursuit of self-interest and the enhancement of market environment pressure given by criminal law-based copyright protection are transformed into internal and external pressure to stimulate the ES and promote the further strengthening of ES (Ganotakis et al., 2021). Innovation is the most direct embodiment of ES, and it is the driving force of economic growth and guarantees stable economic growth. Innovation is always regarded as high investment and high-risk behavior. Entrepreneurs with high ES are more sensitive to the market environment, dare to take risks, and pioneer in consumer markets through scientific and technological innovation. Then, entrepreneurs turn enterprise innovation into actual productivity to promote economic development (Popoola, 2021). Currently, the continuous strengthening of market competition and the limitation of enterprise resources make it difficult for most enterprises to carry out and transform innovation activities and achievements. It forces enterprises to cooperate to complete innovation activities. Through cooperation, enterprises can share the information flows and resources, reducing the cost of innovation investment. Moreover, the innovation results produced by enterprise cooperation have a positive role in promoting and driving the region’s economy (Yang et al., 2021). Besides, strong copyright protection prevents innovative achievements from being copied. As a result, entrepreneurs disclose their proprietary information, reducing research costs, avoiding repeated investment, further stimulating ES, forming an innovation linkage effect in the industry, and promoting economic development through industrial progress (Sudartianto et al., 2021).

Based on this, the following hypothesis is put forward:

**H3:** ES plays an intermediary role between criminal law-based copyright protection and economic growth.

**FIGURE 3:**
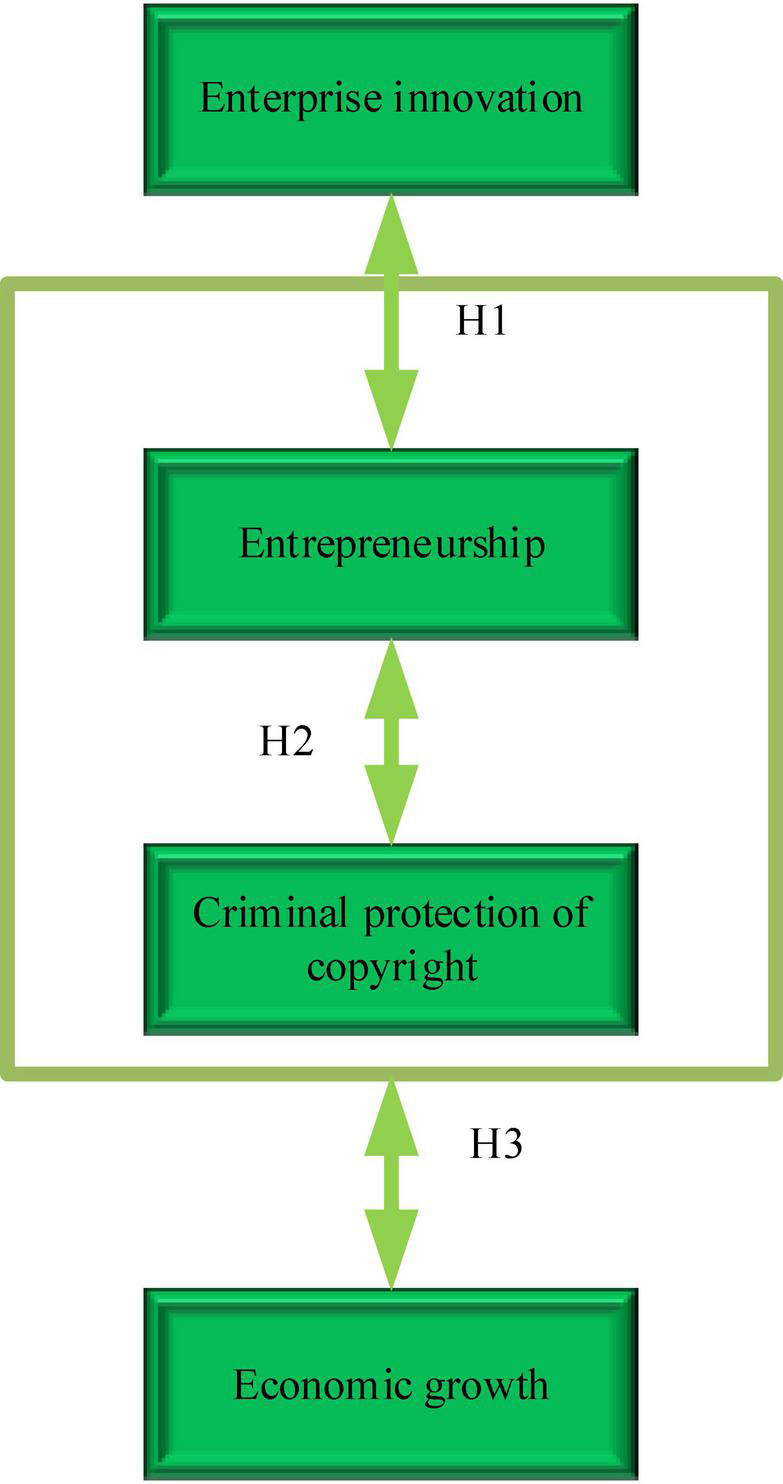
The relationship between economic growth, ES, enterprise innovation, and the strength of criminal law-based copyright protection.

Based on the above hypotheses, Figure 3 is the model given.

### Questionnaire design and data processing

#### Selection of scale

The data are collected by designing a questionnaire survey. The scale is adjusted according to the current situation of each variable. In addition, it mainly measures the ES scale, enterprise innovation strength scale, criminal law-based copyright protection scale, and economic growth scale and further gives the questionnaire structure. Table 1 shows the composition of the questionnaire.

**TABLE 1:** Composition of the questionnaire.

Serial number of the scale	1	2	3	4
Specific contents of the scale	ES	Enterprise innovation strength	Criminal law-based copyright protection	Economic growth

#### Index division and data source

Given the accessibility of data, the enterprise R&D cost is used to measure enterprise innovation. The number of employees owned by individual and private enterprises to the total employed population is used to measure ES. Besides, the data on the strength of criminal law-based copyright protection is from the *China Marketization Index*: *2011 Report on the Relative Process of Marketization in Various Regions* prepared by Fan Gang and Wang Xiaolu. Finally, it takes “gross domestic product (GDP)” as a measure of economic growth.

#### Data processing and scale inspection

Statistical Product and Service Solutions (SPSS) platform is used to make descriptive statistics and questionnaire reliability analysis on the variable data of the questionnaire. The Chi-square test is used to judge the robustness of the data on each scale. Meanwhile, the SPSS platform is used to test the reliability and validity of each scale by Kaiser–Meyer–Olkin (KMO) value and Barrett’s test.

### Test model and variable description

#### Establishment of the H1 model

The following model is constructed to test H1.

(1) $$R\,D\,i\,n\,t_{i,t}=\alpha +\beta_{1}\,E_{i,t}+\beta_{2}\,C\,o\,n\,t\,r\,o\,l_{i,t}+\beta_{3}\,I\,n\,d\,D\,u\,m\,m\,y_{i,t}$$

$$+\beta_{4}\,T\,i\,m\,e\,D\,u\,m\,m\,y_{i,t}+\varepsilon_{1}$$

Here, *RDint* represents the R&D strength of the enterprise, ***E*** represents the ES, *IndDummy* represents the enterprise type, *TimeDummy* represents the year, and ε_*1*_ is the residual term. Table 2 is the definition of the relevant variables of the model.

**TABLE 2:** The variable distribution of the H1 model.

Variable meaning	ES	Strength of enterprise innovation	Economic growth
Type	Intermediate variable	Independent variable	Dependent variable
Variable interpretation	Number of patents (piece)	R&D cost (10,000 RMB)	Regional GDP (100 million RMB)

#### Establishment of H2 and H3 models

The three-step method given by Baron and Kenny is used to test the intermediary role of ES, and H2 and H3 are verified simultaneously. A regression model is built for that:


(2) $$G\,D\,P_{i\,t}=\beta_{0}+\beta_{1}\,I\,P\,R\,S_{i\,t}+\mu_{i}+\mu_{t}+\varepsilon_{i\,t}$$

(3) $$E_{i\,t}=\beta_{2}+\beta_{3}\,I\,P\,R\,S_{i\,t}+\mu_{i}+\mu_{t}+\varepsilon_{i\,t}$$

(4) $$G\,D\,P_{i\,t}=\beta_{4}+\beta_{5}\,I\,P\,R\,S_{i\,t}+\beta_{6}\,E_{i\,t}+\mu_{i}+\mu_{t}+\varepsilon_{i\,t}$$

(5) $$G\,D\,P_{i\,t}=\beta_{7}+\beta_{8}\,I\,P\,R\,S_{i\,t}+\beta_{9}\,E_{i\,t}+\sum \beta_{j}\,X_{i\,t}+\mu_{i}+\mu_{t}+\varepsilon_{i\,t}$$

*GDP* represents the regional GDP, *IPRS* represents the strength of criminal law-based copyright protection, and μ_*i*_ represents the time unchanged and unobserved provincial characteristic factors, such as the geographical location, resource endowment, and innovative culture of each province. The exertion of ES is also affected by national policies, laws, and other factors that change over time. Therefore, the time effect μ_*i*_ is also considered when estimating the model. ε_*it*_ is the residual term and *X*_*it*_ is the controlled variable. The panel fixed-effect model estimates all models. Table 3 shows the definitions of relevant variables.

**TABLE 3:** The variable distribution of H2 and H3 models.

Variable meaning	ES	Strength of criminal law-based copyright protection	Economic growth
Type	Intermediate variable	Independent variable	Dependent variable
Variable interpretation	Number of patents (piece)	The proportion of market turnover in GDP (%)	Regional GDP (100 million RMB)

After the estimated regression model is determined, SPSS estimates and calculates the coefficient and residual term of each variable in the model. Then, the following empirical analysis continues.

## Empirical analysis results

### Descriptive statistics

A questionnaire survey is conducted in various enterprises in some regions of China. A total of 200 questionnaires are distributed to enterprise middle and senior leaders and company directors. Then, 185 valid copies are recovered, with a 92.5% effective recovery rate, meeting the requirements of the questionnaire survey.

**FIGURE 4:**
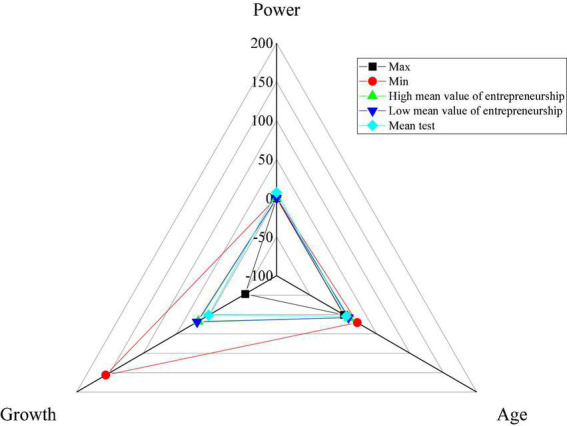
Descriptive statistical results of enterprise innovation.

Figure 4 shows the descriptive statistical results of enterprise innovation.

Figure 4 reveals that enterprises with different ES indexes have different investments in innovation. Moreover, the enterprises with a higher ES index invest more in innovation than those with a lower ES. The regional economic growth increases with the enterprise’s innovation strength. The above shows that the H1 proposed has been verified.

**FIGURE 5:**
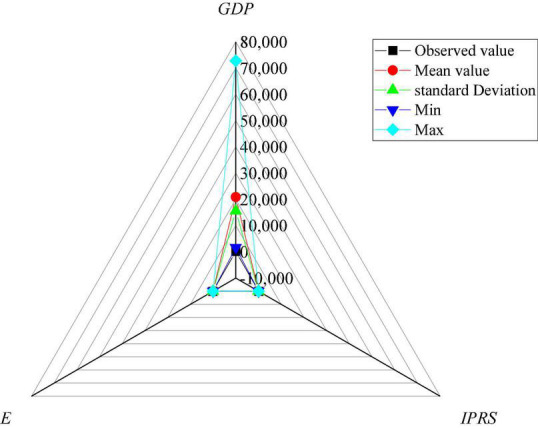
Descriptive statistical results of economic growth.

Figure 5 shows the descriptive statistical results of economic growth.

Figure 5 suggests great differences and imbalances in criminal law-based copyright protection, economic growth capacity, and ES among regions in China. However, the annual data research results show that the strength of criminal law-based copyright protection and ES have been significantly developed in most regions. Besides, the ability of economic growth has also developed rapidly. The above results preliminarily verify the proposed hypothesis.

### Analysis of model regression results

#### Model regression results of H1

**FIGURE 6:**
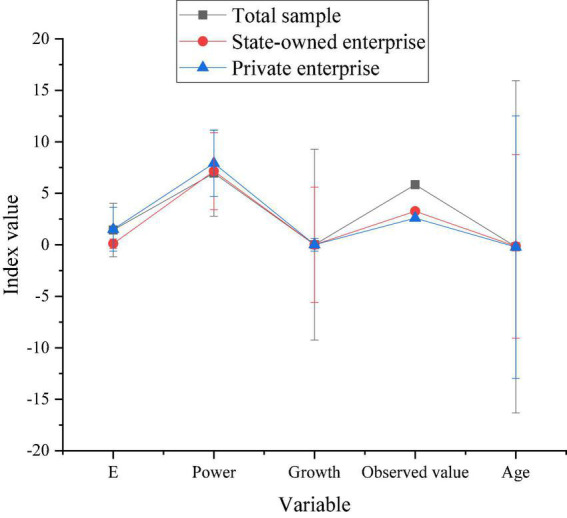
Model regression results of H1.

Figure 6 shows the model regression results of H1.

Figure 6 reveals that the significance of ES under the 1% index is greater than 0. In other words, ES significantly impacts the intensity of enterprise innovation investment. Moreover, enterprise innovation is also increasing with the continuous improvement of ES. It also shows that entrepreneurs with a strong ES mostly choose to take risks in innovation and uncertainty to obtain high returns. The results confirm the H1 theory.

### Model regression results of H2 and H3

**FIGURE 7:**
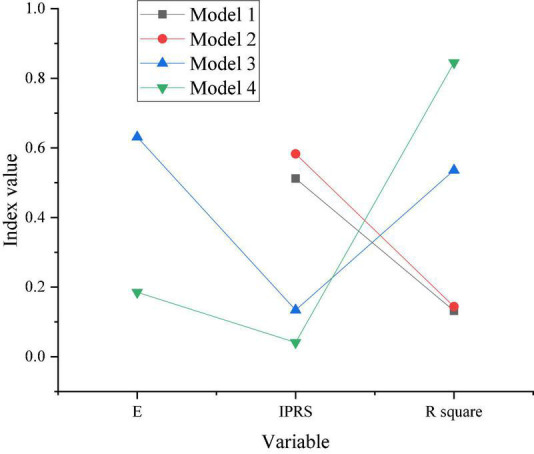
The model regression results of H2 and H3.

Figure 7 shows the model regression results of H2 and H3.

Figure 7 shows that in model 2, the significance of the impact of criminal law-based copyright protection on ES under the 1% index is greater than 0, and the regression coefficient is 0.59. In other words, the greater the criminal law-based copyright protection is, the greater the region’s ES is. It shows that the stronger the criminal law-based copyright protection is, the lower the probability of innovation achievements being stolen is and the more significant the positive impact of ES is. The proposed H2 is verified. In addition, the data of model 3 with ES show that the impact of copyright protection on economic growth is not significant. However, the significance of the impact of ES on economic growth under the 1% index is greater than 0, and the regression coefficient is 0.63. It shows that the impact of criminal law-based copyright protection on economic growth is not directly reflected but indirectly affects economic growth by using the intermediate variable of ES. The H3 proposed is verified.

### Reliability and validity analysis of the scale

The SPSS platform is adopted to test the reliability and validity of each scale. Table 4 shows the inspection results:

**TABLE 4:** Reliability and validity test results of each scale.

Scale name	Kaiser–Meyer–Olkin measure value	Bartlett’s test value		
		Chi-square	Degree of freedom	Significance probability value
ES	0.85** (1.44)	729.47	25	0.00
Strength of enterprise innovation	0.92** (2.12)	945.45	64	0.00
The strength of criminal law-based copyright protection	0.89** (1.57)	831.87	65	0.00
Economic growth	0.89** (0.45)	855.16	39	0.00

**Significant meaning.

Table 4 shows that Barrett’s test of the ES scale, KMO value, significance probability, and the degree of freedom are 729.47, 0.85, *P* = 0.00 < 0.05, and 25, respectively. The result proves that the ES scale has high reliability. Those indexes of the enterprise innovation scale are 945.45, 0.92, *P* = 0.00 < 0.05, and 64, respectively. It indicates that the scale of the strength of enterprise innovation has high reliability. By comparison, those indexes of the criminal law-based copyright protection scale are 831.87, 0.89, *P* = 0.00 < 0.05, and 65, respectively. It indicates that the scale of criminal law-based copyright protection has high reliability. Finally, the economic growth scale indexes are 855.16, 0.89, *P* = 0.00 < 0.05, and 39, respectively. It indicates that the economic growth scale has high reliability.

### Data robustness analysis

**FIGURE 8:**
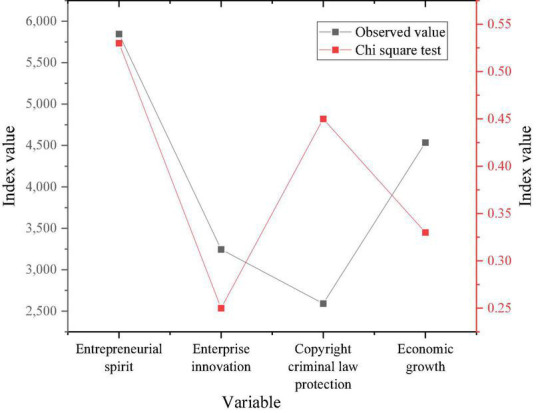
Data robustness test results of each scale.

SPSS analysis software is used to test the robustness of the scale data. Figure 8 shows the results.

Figure 8 reveals that the Chi-square test value of the ES scale is 0.53 > 0.5, and that of the enterprise innovation scale is 0.25 < 0.5. That of the criminal law-based copyright protection scale is 0.45 < 0.5. That of the economic growth scale is 0.33 < 0.5. The above numerical result shows that in the questionnaire designed, except for the slightly unstable ES scale data, the robustness test value of other scales is less than 0.5. Thus, the overall questionnaire data are basically in a stable state. The above shows that the data robustness of the scale provided is basically good and within the controllable range.

### Discussion

The research subject is the people with at least undergraduate degrees and occupying the middle and senior management positions in enterprises. During the survey, the stronger the criminal law-based copyright protection is, the more they tend to invest more resources in enterprise innovation. The decisions they make in this process tend to be more rational and decisive. That is to say, in the continuous improvement of copyright protection, the higher the managers’ ES is, the more rational their decisions are. As Koray (2021) said, the high-intensity criminal law-based copyright protection only reduces the possibility of stealing technological innovation and temporarily protects the legal monopoly compensation. Even if the copyright is infringed, higher compensation can be generated to ensure the status of innovative products from the institutional level. At the same time, protection of copyright also strengthens the subdimensions of ES: adventurous spirit and desire for success, thus improving the ES. Additionally, Williams et al. (2021) also point out that criminal law-based copyright protection protects the legitimate rights and interests of knowledge creators. Also, it requires creators to disclose their content to the society when applying for criminal law protection of copyright, giving the public the thinking of re-innovation and optimization. The copyright owners turn knowledge into commodities and ensure the correct use of the copyright. In doing so, they enhance the labor production efficiency, protects and stimulates the entrepreneurs’ responsibility to serve society, and promotes the cultivation of entrepreneurs’ ES.

## Conclusion

Literature collection and empirical analysis are used to study the relationship between criminal law-based copyright protection and ES. Moreover, variables such as enterprise innovation and economic growth are introduced to discuss their relationship. First, the concepts of ES, criminal law-based copyright protection, and enterprise innovation are given. Next, hypotheses are put forward, and relevant models are established according to the concept and relevant literature. Finally, the hypotheses are tested by setting experiments and empirical analysis. The empirical analysis shows that (1) the higher entrepreneurs’ ES is, the greater the innovation strength of enterprises; (2) criminal law-based copyright protection has a significant positive impact on ES; (3) ES plays an intermediary role in the relationship between criminal law-based copyright protection and economic growth. (4) After testing, the scale’s reliability, validity, and robustness are good, showing that the scale indexes and the data source are reliable. The theoretical significance of this exploration is to provide a reference for the further study of the relationship between ES and criminal law-based copyright protection. The practical significance is to study the relationship between ES and criminal law-based copyright protection and then find ways to improve ES based on this result. The deficiency is that few indexes are selected for the scale, and there is a certain error. It is expected to enrich the index to make the experimental results more accurate. This exploration aims to study the relationship between criminal law-based copyright protection and ES to provide a reference for further optimizing criminal law-based copyright protection.

## Data availability statement

The raw data supporting the conclusions of this article will be made available by the authors, without undue reservation.

## Ethics statement

The studies involving human participants were reviewed and approved by the Tsinghua University Ethics Committee. The patients/participants provided their written informed consent to participate in this study. Written informed consent was obtained from the individual(s) for the publication of any potentially identifiable images or data included in this article.

## Author contributions

The author confirms being the sole contributor of this work and has approved it for publication.
