# Supplementary material for: Criminal law-based copyright protection with entrepreneurial spirit
Source: Front Psychol. 2022 Sep 8;13:944122. doi: 10.3389/fpsyg.2022.944122 (PMC9493109; doi:10.3389/fpsyg.2022.944122)
Supplement: Supplementary file 1 [file Data_Sheet_1.docx]

Appendix (questionnaire details):

Part one: basic information

The following is a description of your personal and business situation. Please choose the corresponding options.

1. Gender

□Male □Female

2. Age

□Below 18 □18-24 □25-30 □31-40 □Over 41

3. Working age

□Below 5 years □5-10 years □11-20 years □Over 21 years

4. Educational background

□Junior college or below □Bachelor □Master □Doctor

5 Post

□Employee □Team leader □Middel manager □Senior executive

6. Enterprise nature

□State-owned □Private □Foreign proprietorship □Sino-foreign joint venture □Other

7 Maximum employee number

□Below 100 □101-200 □201-300 □301-400 □Over 400

8 Enterprise operation years

□0-5 years □6-10 years □11-15 years □16-20 years □Over 20 years

Part two: Entrepreneurial Spirit

Please select the corresponding number according to your actual situation and the company.

| Item content | Strongly disagree | Disagree | General | Agree | Strongly agree |
| --- | --- | --- | --- | --- | --- |
| I actively develop and introduce new products or services. | 1 | 2 | 3 | 4 | 5 |
| I actively explore new technologies and methods to develop the enterprise's business. | 1 | 2 | 3 | 4 | 5 |
| I actively look for new resources and markets to help enterprises develop business. | 1 | 2 | 3 | 4 | 5 |
| I actively innovate the enterprise's process, system, and management method. | 1 | 2 | 3 | 4 | 5 |
| I actively encourage and support employees to innovate in all aspects. | 1 | 2 | 3 | 4 | 5 |
| I actively promote the growth of innovation investment of the company. | 1 | 2 | 3 | 4 | 5 |
| I prefer higher risks over low-risk and low-return projects for higher returns | 1 | 2 | 3 | 4 | 5 |
| I tend to take bold, positive, and rapid actions to achieve the set goals. | 1 | 2 | 3 | 4 | 5 |
| When making decisions in the face of uncertainty, I tend to take a bold and positive attitude to seize opportunities. | 1 | 2 | 3 | 4 | 5 |
| Compared with "deep cultivation and meticulous work" in existing fields, I tend to explore and open up new fields. | 1 | 2 | 3 | 4 | 5 |
| I often take the initiative to review the development trend of the industry to grasp the opportunity and take action early. | 1 | 2 | 3 | 4 | 5 |
| I often take the initiative to formulate strategies that will change the market. | 1 | 2 | 3 | 4 | 5 |
| I often introduce new business models or business concepts. | 1 | 2 | 3 | 4 | 5 |
| I often lead enterprises to introduce new products/services into the market first or take the lead in introducing the latest management mode and new technology. | 1 | 2 | 3 | 4 | 5 |
| I often take action before my competitors | 1 | 2 | 3 | 4 | 5 |

Part three: Enterprise innovation

Please select the corresponding number according to your actual situation and the company.

| Item content | Strongly disagree | Disagree | General | Agree | Strongly agree |
| --- | --- | --- | --- | --- | --- |
| The company has a large number of patent applications. | 1 | 2 | 3 | 4 | 5 |
| The company has a high success rate in developing new products or services. | 1 | 2 | 3 | 4 | 5 |
| The development and transformation of new products or services of the company are fast. | 1 | 2 | 3 | 4 | 5 |
| Our innovative products and services can open up new markets. | 1 | 2 | 3 | 4 | 5 |
| The proportion of the company's innovative products and services in the total products continues to increase. | 1 | 2 | 3 | 4 | 5 |
| The market share of our innovative products and services continues to increase. | 1 | 2 | 3 | 4 | 5 |
| The proportion of the sales of the company's innovative products and services in the total sales continues to increase. | 1 | 2 | 3 | 4 | 5 |
| The proportion of the profit of the company's innovative products and services to the total profit continues to increase. | 1 | 2 | 3 | 4 | 5 |

Part four: Enterprise copyright protection

| Item content | Strongly disagree | Disagree | General | Agree | Strongly agree |
| --- | --- | --- | --- | --- | --- |
| The company has established relevant departments for the protection of innovative products. | 1 | 2 | 3 | 4 | 5 |
| The company often provides learning opportunities for personnel in the product management department of the enterprise. | 1 | 2 | 3 | 4 | 5 |
| The company often inspects the product management department of the enterprise. | 1 | 2 | 3 | 4 | 5 |
| The company attaches great importance to enterprise products. | 1 | 2 | 3 | 4 | 5 |

Part five: The relationship between entrepreneurial spirit and copyright protection

| Item content | Strongly disagree | Disagree | General | Agree | Strongly agree |
| --- | --- | --- | --- | --- | --- |
| The company's leaders often publish meetings to require that the copyright protection of enterprise products must be in place. | 1 | 2 | 3 | 4 | 5 |
| The company's leaders will regularly verify the copyright protection of the enterprise's products with the relevant government departments. | 1 | 2 | 3 | 4 | 5 |
